# Calcium butyrate efficacy in pediatric irritable bowel syndrome: Randomized placebo‐controlled multiomics‐based clinical trial

**DOI:** 10.1002/jpn3.70154

**Published:** 2025-07-09

**Authors:** Fernanda Cristofori, Francesco Maria Calabrese, Ilaria Iacobellis, Monica Santamaria, Giuseppe Celano, Ilario Ferrocino, Emanuela Di Sabato, Rocco Pergola, Vanessa N. Dargenio, Leonardo Paulucci, Maria De Angelis, Ruggiero Francavilla

**Affiliations:** ^1^ Interdisciplinary Department of Medicine, Pediatric Section, Children's Hospital’ Giovanni XXIII’ University of Bari Aldo Moro Bari Italy; ^2^ Department of Soil, Plant and Food Sciences University of Bari Aldo Moro Bari Italy; ^3^ Department of Agricultural, Forest and Food Sciences University of Turin Grugliasco Italy

**Keywords:** 16S sequencing, fecal metabolomics, gut homeostasis, intestinal microbiota, SCFAs

## Abstract

**Objective:**

Irritable bowel syndrome (IBS) is one of the most common functional gastrointestinal disorders, and treatment involves nonpharmacological and pharmacological therapies, even if there is no optimal therapy. This randomized, placebo‐controlled, double‐blind trial aimed to evaluate the efficacy of calcium butyrate supplementation in reducing IBS symptoms and to assess its effects on gut microbiota composition and relative metabolic profiles through a multiomics approach.

**Methods:**

Children aged 4–17 years with IBS diagnosed according to the Rome IV criteria were randomized to receive either a formulation based on calcium butyrate (500 mg/day) or placebo for 8 weeks, followed by a 4‐week follow‐up period. Clinical assessments included the visual analogue scale (VAS) and gastrointestinal symptom rating scale (GSRS). Fecal samples were analyzed via 16S metataxonomics and targeted/untargeted metabolomics. The primary outcome was an ≥50% reduction in the VAS scores. Secondary outcomes included microbiota composition changes and metabolite profile alterations.

**Results:**

Fifty‐one children were enrolled. Treatment success was significantly higher in the butyrate group (73% vs. 3.8%, *p* < 0.0001). VAS and GSRS scores were significantly reduced in butyrate‐treated patients at the end of treatment and postwashout. Metataxonomic analysis revealed increased short chain fatty acids‐producing bacteria, including Lachnospiraceae and *Ruminococcus gauvreauii*, while pro‐inflammatory taxa such as *Ruminococcus gnavus* decreased. Metabolomics confirmed significant changes in SCFA and VOCs, supporting microbiota modulation.

**Discussion:**

Calcium butyrate supplementation effectively reduced IBS symptoms and induced beneficial microbiota and metabolic shifts in pediatric patients. These findings support butyrate as a potential therapy in pediatric IBS, warranting further large‐scale investigations to confirm efficacy and optimize dosing strategies.

**Clinical Trial Identification Number:**

Study registered on: https://ClinicalTrials.gov. Number of registration: NCT04566679 Date of registration: 28/09/2020. Date of first enrollment of patients: 01/05/2021.

## Abstract (highlights)

**What is Known:**

- Butyrate is a short‐chain fatty acid with anti‐inflammatory properties and a key role in maintaining gut homeostasis.
- Clinical studies in adults suggest that butyrate supplementation can alleviate irritable bowel syndrome (IBS) symptoms and influence gut microbiota composition.
- There is a lack of evidence on the efficacy and safety of butyrate supplementation in pediatric IBS.

**What is New:**

- This is the first randomized, double‐blind, placebo‐controlled trial evaluating butyrate in pediatric IBS.
- Butyrate supplementation significantly improved IBS symptoms, with a higher treatment success rate than placebo.
- Multiomics analysis revealed beneficial microbiota and metabolite shifts, supporting its potential therapeutic role in pediatric IBS.

## Graphical abstract

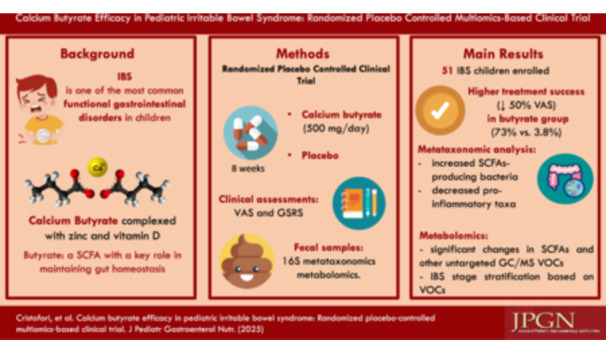

## 1 INTRODUCTION

Irritable bowel syndrome (IBS) is a functional gastrointestinal disorder (FGID) that significantly impacts patients' quality of life (QoL) and social well‐being. Characterized by abdominal pain, distension, bloating, diarrhea, and constipation,^1^ its diagnosis relies on the Rome IV criteria.^2^ IBS can be classified into four subtypes based on predominant clinical symptoms: diarrhea‐predominant (IBS‐D), constipation‐predominant (IBS‐C), mixed stool pattern (IBS‐M), and unclassified (IBS‐U).^3^ Management of IBS typically involves a multidisciplinary approach encompassing nonpharmacological and pharmacological therapies. However, these interventions often do not target the underlying causes, which remain poorly understood.

Several pathophysiological mechanisms have been implicated in IBS, including abnormal gastrointestinal (GI) motility, visceral hypersensitivity, low‐grade inflammation, and altered brain–gut interactions. Emerging evidence suggests that genetic and environmental factors, such as dietary habits and gut microbiota resilience, play critical roles in IBS pathophysiology.^4^, ^5^

Microbiota composition studies revealed significant differences between IBS patients and healthy controls. For instance, IBS‐D patients exhibit increased levels of *Enterobacteriaceae* and decreased abundance of *Faecalibacterium prausnitzii*, indicating an imbalance between beneficial and potentially harmful gut bacteria.^6^ Additionally, other studies reported increased levels of *Lactobacillus* and *Ruminococcus* and decreased levels of *Bifidobacterium* and *Faecalibacterium*.^7^ These microbial shifts are often associated with increased intestinal permeability, inflammation, and reduced short‐chain fatty acids (SCFAs) production.^8^

SCFAs, mainly acetate, propionate, and butyrate, are organic acids produced by bacterial fermentation of undigested dietary carbohydrates and are essential for maintaining intestinal health. Butyrate, in particular, serves as the primary energy source for colonocytes and has been shown to support colon mucosa health by promoting epithelial cell differentiation, turnover, and viability.^9^, ^10^, ^11^ Notably, reduced butyrate levels have been observed in IBS patients compared to healthy controls.^12^

While evidence support the efficacy of butyrate supplementation in alleviating IBS symptoms in adults,^13^, ^14^, ^15^ data on its use in pediatric cohorts are not available.

This study is aimed at evaluating whether calcium butyrate supplementation can relieve IBS symptoms in pediatric patients and to assess its impact on gut microbiota composition, function, and metabolite profiles through a multiomics approach.

## 2 METHODS

We report data on a randomized double‐blind, placebo‐controlled, parallel‐group trial conducted at the Pediatric Gastroenterology Department of the University of Bari from 2021 to 2023. Fecal metaomics analyses have been performed at Department of Soil, Plant and Food Sciences, of the University of Bari.

### 2.1 Ethics statement

The protocol was approved by the Institutional Ethical Committee (Approval Number: 0045/6515, January 14, 2021), and the trial was registered on ClinicalTrials.gov (Registration Number: NCT04566679). Written informed consent was obtained from all participating children's parents or legal guardians before enrollment.

### 2.2 Eligibility of patients

Children aged 4–17 years diagnosed with IBS according to the Rome IV Criteria were consecutively enrolled. Exclusion criteria: antibiotic or probiotic use within the previous 2 months, growth failure or malnutrition, prior abdominal surgery, GI comorbidities (e.g., inflammatory bowel disease, celiac disease, *Helicobacter pylori* infection), lactose intolerance, difficulty in swallowing tablets.

### 2.3 Study design

The study comprised three phases: a 2‐week run‐in period (Weeks 1–2), an 8‐week treatment period (Weeks 3–10), and a 4‐week follow‐up period (Weeks 11–14). Four study visits were scheduled:

− Visit 1 (Enrolment): Confirm diagnosis and eligibility criteria, explain study procedures, sign informed consent, and provide instructions on diary completion and stool sample collection.
− Visit 2 (end of run‐in T0): Clinical assessment; according to the recommendation for trials for FGIDs,^16^, ^17^ only patients with persistent symptoms during the run‐in period (visual analogue scale [VAS] greater than or equal to three) were eligible to proceed. Randomization and product allocation were performed, and stool samples were collected.
− Visit 3 (end of treatment T1): Clinical assessment and stool sample collection.
− Visit 4 (end of follow‐up T2): Clinical assessment and stool sample collection.

### 2.4 Intervention

At Visit 2, children were randomly assigned, using a computer‐generated randomization list, to receive either oral calcium butyrate (500 mg) supplemented with zinc (5 mg) and vitamin D (500 IU) in functional release tablets (named throughout the text and in the figures as butyric acid [BA]) or placebo, administered once daily for 8 weeks. Both the active product and placebo tablets were identical in shape, size, taste, and appearance and were provided by Difass International to ensure blinding for investigators and participants.

### 2.5 Compliance and monitoring

Compliance was assessed by counting returned tablets, with noncompliance defined as missing more than 20% of doses. Adverse events were documented throughout the study. Participants were instructed to avoid dietary changes and prohibited from consuming probiotics or prebiotics outside of the study protocol.

### 2.6 Clinical data collection

Throughout the study, all symptoms were documented in a patient diary. To assess symptom severity, all children daily completed a combination of the self‐reporting VAS with the faces pain scale to facilitate children's understanding. The 0–10 mm VAS scale (0 no pain, 10 worst possible pain) included a horizontal color gradient (green–red), while the faces pain scale comprises six faces ranging from a relaxed face to a face showing intense pain. Children indicated their pain level by pointing to a position on the scale and drawing a line to mark it.^18^

Participants completed the gastrointestinal symptom rating scale (GSRS) every 2 weeks, which evaluates the severity of various GI symptoms. The GSRS includes a score ranging from 0 (no pain) to 3 (severe pain) for the following symptoms: abdominal pain, heartburn, regurgitation, fatigue, nausea and vomiting, borborygmi, abdominal distension, belching, increased flatulence, altered fecal transit, stool consistency, feeling of incomplete evacuation, and urgency to defecate.^19^

### 2.7 Outcome measures

The primary outcome was to investigate whether BA supplementation, compared with placebo, can decrease by at least 50% VAS (treatment success).^17^

Secondary outcomes were (i) improvement in GI symptoms at the end of treatment and follow‐up, as assessed by the VAS and GSRS; (ii) change in microbiota composition, function, and metabolite profiles.

### 2.8 Fecal DNA sample extraction

DNA extractions were performed on fecal samples in triplicate at three time‐points: before and at the end of treatment and after follow‐up. The extraction steps followed the procedure reported previously.^20^

### 2.9 PCR amplification and sequencing

Variable V3–V4 region of the 16S ribosomal RNA (rRNA) was sequenced on an Miseq. 2 Illumina platform available as our department facility.

PCR amplicons obtained by using the universal Illumina primer couple (https://support.illumina.com/documents/documentation/chemistry_documentation/16s/16s-metagenomic-library-prep-guide-15044223-b.pdf) were purified using an Agencourt AMPure kit (Beckman Coulter) and labeled using the Nextera XT index kit (Illumina Inc.) according to manufacturer's instructions. A concentration of 4 ng/µL for each sample library was obtained before pooling.

### 2.10 Taxonomic analysis

PCR primers and Illumina adapters were removed using the Cutadapt tool.^21^ The sequence quality was assessed using FastQC^22^ and MultiQC.^23^ Reads were denoised through the Qiime2 DADA2 denoise‐paired plugin.

A V3–V4 specific classifier, was built up starting from SILVA release 138. The QIIME2‐compliant classifier was obtained by low‐quality sequence removal, dereplication and a filtering steps based on length and taxonomy (primer couple 341 F/805 R). Unassigned mitochondrion and chloroplast ASVs were removed. Diversity metrics were computed by using QIIME2 nested plugins.

### 2.11 Volatile organic compounds (VOCs) and SCFA from targeted metabolomics

An aliquot of 1 g per each fecal sample was used to run fecal metabolomics.

Gas chromatography‐mass spectrometry (GC‐MS) analysis was performed using a Clarus 680 (Perkien Elmer) equipped with an Rtx‐WAX capillary column (30 m × 0.25 mm i.d., 0.25 μm film thickness) (Restek). Column applied parameters and other details were previously reported.^24^ The gas chromatography system was coupled with a Clarus SQ 8C single quadrupole mass spectrometer (Perkien Elmer).

Targeted GC‐MS analyses measured acetic, propanoic, butanoic, isobutyric, and isovaleric acids concentrations.

### 2.12 Statistical analyses

To show the effectiveness of BA, assuming a 20% placebo effect and a minimum 35% difference in response, with a study power of 80% and a *p*‐value of 0.05, 23 patients per group were needed. Considering a 10% dropout rate, this number was increased to 25.

The *χ* ^2^ test or the Fisher exact test was used to compare percentages and nominal variables. For continuous variables, differences between patients in the two treatment arms were compared using an analysis of variance (ANOVA), whereas the Wilcoxon test was used to compare the mean values. All statistical tests were two‐tailed and performed at the 5% significance level. The statistical analyses were performed using the JMP SAS Institute program version 9.

Treatment success was defined as a decrease in abdominal pain intensity after treatment of at least 50% from baseline.^17^

The complete matrices of genus and VOC abundances were inspected using discriminant analysis of principal component (DAPC) using the R “adegenet” package v2.1.1.^25^ The a priori hypothesis was inspected without superimposing any metadata grouping condition and using the find clusters clustering algorithm. Metabolic pathway predictions were obtained from 16S rRNA abundance matrix using Picrust2 software.^26^ A BH‐corrected Welch test and a fold change analysis were run between the thesis groups to retrieve significant changes in taxa, biochemical pathways, and VOCs. Statistically significant variables in the pairwise group comparisons were graphically rendered as a volcano plots.

## 3 RESULTS

Forty‐four out of 95 potential participants screened at the first visit were excluded; 25 of these did not meet inclusion criteria, 10 declined to participate, and nine experienced symptom improvement during the run‐in phase. Demographic characteristics of the study cohort are summarized in Table 1.

**Table 1:** Demographics data of the enrolled patient.

	Calcium butyrate (*n* = 25)	Placebo (*n* = 25)	*p*‐values
Gender (males %)	65.3%	54.1%	NS
Age	12.6 ± 2.6	12.7 ± 3.3	
Mean ± SD (95% CI)	(95% CI: 11.5–13.7)	(95% CI: 11.5–13.8)	NS
IBS‐subtipes (*n*)			
IBS‐C (constipation)	8	7	NS
IBS‐D (diarrhea)	6	5	NS
IBS‐M (mixed)	3	3	NS
IBS‐U (unsubtyped)	9	10	NS
Familiarity for IBS	26%	24%	NS
BMI (mean ± SD)	19.8 ± 3.2	21.3 ± 4.4	NS
VAS (before treatment)	6.07 ± 1.3	4.6 ± 1.3	*p* < 0.01
Mean ± SD (95% CI)	(5.5–6.6)	(4.1–5.2)	
GSRS (before treatment)	13.1 ± 2.7	12.5 ± 4.3	NS
Mean ± SD (95% CI)	(12.8–15)	(10.7–14.3)	

Abbreviations: BMI, body mass index; CI, confidence interval; GSRS, gastrointestinal symptom rating scale; IBS, irritable bowel syndrome; NS, not significant; SD, standard deviation; VAS, visual analogue scale.

Fifty‐one patients were randomized into two study groups: 26 participants received BA, and 25 received the placebo. One participant discontinued the study due to difficulty swallowing the tablets. No adverse events/side effects were reported in either the BA or placebo groups.

**Figure 1:**
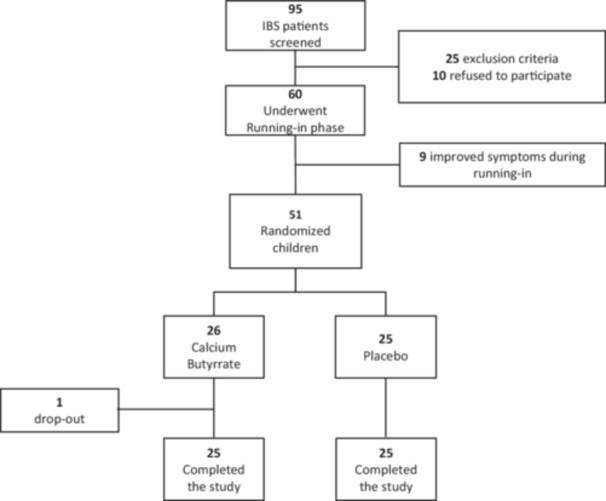
Patients flow‐chart. IBS, irritable bowel syndrome.

Figure 1 provides an overview of participant flowchart throughout the trial, from eligibility assessment to the follow‐up phase. At the final assessment, clinical data were available for 50 of the 51 randomized participants.

### 3.1 Stool samples

Stool samples were available for 14 patients. However, one sample from each group was excluded due to insufficient DNA quantity. Additionally, some aliquots were too low for GC/MS.

As a result, metabolite profile analyses were performed on a total of 55 samples distributed across the study groups as follows:

- Baseline (pretreatment T0): 12 samples from the BA and 11 samples from the placebo group.
- End of treatment (T1): 7 samples from the BA group and 7 from the placebo group.
- Postfollow‐up (T2): 8 samples from the BA group and 10 from the placebo group.

### 3.2 Primary outcome

Treatment success was significantly higher in patients receiving butyrate, as compared with placebo, at both intention‐to‐treat (73% vs. 3.8%; *p* < 0.0001) and per protocol (76% vs. 8%; *p* < 0.0001) analysis, according to our data, two patients need to be treated to reach treatment success in 1 (number needed to treat: 2).

### 3.3 Secondary outcomes

#### 3.3.1 Symptomatic scores

As shown in Table 1, by chance, we found a significantly higher value of the VAS and GSRS scores at baseline in patients who received butyrate compared to the placebo group; therefore, we decided to present the data expressed as variation over the pretreatment value. Supporting Information S1: Table S1 summarizes the VAS and GSRS scores at the different time points in both groups.

When expressed as variation over the pretreatment value (ΔVAS and ΔGSRS), there was a significant decrease of VAS and GSRS both at the end of treatment [ΔVAS:−66.3 ± 23.3% (95% confidence interval [CI]: −75.9 to −56.7%) versus −3.2 ± 33.7% (95% CI: −17.1% to 10.7%) *p* < 0.0001; ΔGSRS: −51.1 ± 26.7% (95% CI: −62.1 to −40.1%) versus −9.2 ± 37.2% (95% CI: −24.5% to 6.2%) *p* < 0.0001] and after follow up (ΔVAS: −78.4 ± 21.5% [95% CI: −87.3 to −69.5%] versus −32 ± 37.4% [95% CI: −47.5% to 16.6%] *p* < 0.0001; ΔGSRS: −60.6 ± 31.5% [95% CI: −73.7 to −47.6%] versus −20.1 ± 37.4% [95% CI: −47.5% to −16.6%] *p* < 0.0001).

#### 3.3.2 Metataxonomics and metabolomics

Both metataxonomics annotated taxa and VOCs have been used to infer the effect of BA treatment impact on intestinal microbiota. With the aim of detecting a possible cluster in DAPC, metabolites from untargeted metabolomics were evaluated based of GSRS and VAS scores.

We also used predicted metabolic pathways to corroborate the presence of VOC.

#### 3.3.3 Alpha and beta diversity estimates

Samples were selected based on the indication of the rarefaction curve (Supporting Information S1: Figure S1).

After inspecting taxa through Faith‐PD, Bray‐Curtis, Jaccard, and Shannon metrics, no differences emerged when group stratification was based on timing or treatment.

#### 3.3.4 DAPC clustering analysis

The complete matrix of genus abundances was inspected using discriminant DAPC to understand if the a priori cluster resulting from the Bayesian information criterion (BIC) curve (Supporting Information S1: Figure S2) may reflect the *a posterior* group assignment. Only three groups were a priori predicted by running the find cluster algorithm (Supporting Information S1: Figure S3).

In the DAPC plot butyrate treated samples at T1 seem to be slightly separate from the placebo‐treated and untreated samples. Moreover, butyrate‐treated T2 sample cloud reveals the non‐lasting treatment effect based on the genera distribution. Looking at the statistical weight of most impacting genera (Supporting Information S1: Figure S4) 16 ot these out of a total of 187 (roughly the 8%) included *Coprobacillus*, *Lachnospiraceae UCG‐008*, *Pseudoflavonifractor*, *Lachnospiraceae FCS020 group*, *Raoultibacter*, *Sarcina*, *Candidatus soleaferrea*, *Lachnospiraceae UCG‐001*, *Monoglobus*, *Epulopisicum*, *Gastranaerophilales*, *Butyrricicoccus*, *Lachonospiraceae NC2004 group*, *Streptococcus*, *Akkermansia* and *Agathobacter*.

As supported in the DAPC assigning plot (Supporting Information S1: Figure S5) samples belonging to the BA group at T1 showed a perfect match with the a priori assigned group. The uncertainty of allocation mainly concerned the placebo and the untreated groups.

#### 3.3.5 Pairwise group comparison aimed at detecting statistically significant genera

The pairwise group comparison and DAPC loading plot partially shared their profile in terms of statistically significant genera.

At the genus level the taxa list, sorted by corrected *p*‐value, included three genera that increased in BA treated samples at T1 when compared with placebo, and specifically *NC2004_group (Lachnospiraceae family)*, *Clostridia UCG‐014* and *Ruminococcus gauvreauii group*, whereas *f__Oscillospiraceae_uncultured*, *TM7x*, *Sellimonas*, *Ruminococcus gnavus group*, and *Saccharimonadaceae* decreased (Table 2).

**Table 2:** Statistically significant genera from pairwise Welch multiple test corrected comparisons.

Welch's comparison pairwise group direction	Genus	FC	log2(FC)	FDR (cor. *p*‐val)	−Log_10_(*p*)
Calcium butyrate T1 versus placebo T1	f__Oscillospiraceae_uncultured	0.31067	−1.6865	0.007686	2.1143
	TM7x	0.17308	−2.5305	0.031646	1.4997
	Clostridia_UCG‐014	12.419	3.6345	0.031676	1.4993
	Sellimonas	0.27791	−1.8473	0.032659	1.486
	[Ruminococcus]_gnavus_group	0.26478	−1.9171	0.037768	1.4229
	Saccharimonadaceae	0.12315	−3.0215	0.038313	1.4166
	Lachnospiraceae_NC2004_group	93.228	6.5427	0.047603	1.3224
	[Ruminococcus]_gauvreauii_group	4.4268	2.1463	0.049617	1.3044
Calcium butyrate T1 versus T0	*NC2004_group* (Lachnospiraceae)	50.36	5.6542	0.037931	1.421
“	*Moryella*	0.12259	−3.0281	0.043463	1.3619
Calcium butyrate T2 versus T0	[Ruminococcus]_gnavus_group	10.147	3.343	0.011891	1.9248
“	Paeniclostridium	20.636	4.3671	0.028444	1.546

*Note*: FDR corrected positive and negative log_2_ fold change values indicate increased and decreased taxa (concerning the first member of comparison). Abbreviations: FC, fold change; FDR, false discovery rate.

On the other hand, within the butyrate arm, other two genera that is, *NC2004_group (Lachnospiraceae)* and *Moryella* increased at T1 compared with T0 (Table 2). At the washout, *Ruminococcus_gnavus_group* and *Paeniclostridium*, significantly increased in butyrate patients at T2 compared to T0, while no differences emerged when T1 and T2 were compared.

#### 3.3.6 Fecal untargeted metabolomics: VOC profiles

The complete set of assigned VOCs included 132 VOCs classified as alcohols (13), aldehydes (10), carboxylic acids (45), carboxylic esters (2), fatty acids (2), hydrocarbons (19), indoles (4), ketones (9), terpenes (7) and one lactone (Supporting Information S1: Table S2).

**Figure 2:**
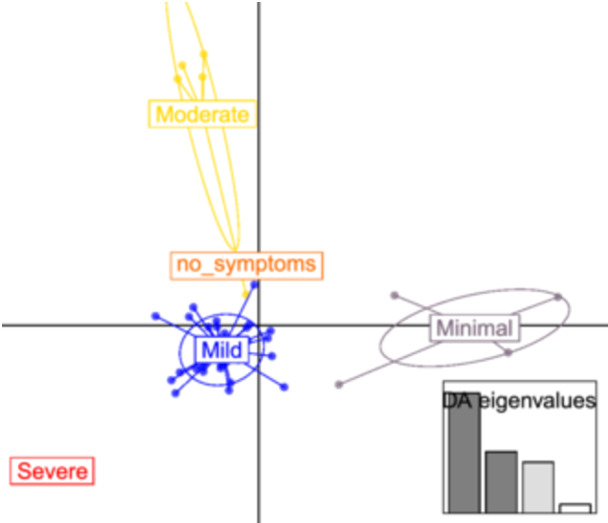
VOC DAPC plot based on GSRS scale classes. The evaluation of GSRS symptoms based on patient administered questionnaires has been used to a posterior cluster sample. Sixty principal components and four eigen values have been used to scatter the DAPC plot. DAPC, discriminant analysis of principal component; GSRS, gastrointestinal symptom rating scale; VOCs, volatile organic compounds.

Trying to better stratify samples, we superimposed the belonging of patients to GSRS classes, based on patient perception of symptom picture, that is, regurgitation, abdominal pain, diarrhea, and constipation. As a result, patients were divided into five groups, from absence to persistence of symptoms (no symptoms, minimal, moderate, mild, and severe). Group ellipsoid placement reflects a meaningful statistical impact (Figure 2).

All clusters appeared as separate in the orthogonal plot, and the most enriched group in terms of samples, composed of patients with mild status, was placed in the third quadrant.

We then investigated the stratification based on timing (untreated or T0, treated or T1 and washout or T2). Although affected by a reduced per group sample number, DAPC succeeded in maximizing the intergroup variance also on this stratification. As a result, mild BA‐treated samples were set apart from the central overlapping cloud batch, and both T1‐treated and T2 washout samples were plotted separately. A slight effect was also detectable in the few patients with minimal symptoms at the washout. In contrast, untreated and placebo samples overlapped in the first quadrant (Supporting Information S1: Figure S6).

#### 3.3.7 Statistically significant VOCs from untargeted metabolomics

When the two arms were compared at T1 Supporting Information S1: Figure S7), a total of seven VOCs showed a significant decrease in butyrate‐treated samples (pentanoic acid, 3‐methylbutyl ester, benzyl alcohol, acetonitrile, 1‐undecanol, 1‐Undecene, 7‐methyl‐, and 9‐Octadecene, [E]‐). In contrast, one has an increased fold change (octanoic acid).

In the BA arm, some VOCs decreased after treatment (T1) if compared with untreated (T0), and precisely: pentanoic acid, 3‐methylbutyl ester, 2‐hexanone, and Ethanol 2‐butoxy‐. No statistically significances emerged from the comparison between T1 and T2 butyrate‐treated samples, whereas when T0 and T2 were compared, 1H‐Pyrrole‐2,5‐dione, 3‐ethenyl‐4‐methyl‐, and 2‐hexanone decreased. Moreover, butanoic acid methyl ester and propanoic acid propyl ester increased at washout.

#### 3.3.8 Predicted biochemical pathways

Delving Picrust2 predictions, in comparison with placebo, BA‐treated samples at T1 showed a decreasing fold change in four different pathways, that is, L‐lysine fermentation to acetate and butanoate, TCA cycleVIII, superpathway of Clostridium acetobutylicum acidogenic fermentation, and pyruvate fermentation to butanoate (Supporting Information S1: Figure S5). Conversely, four pathways were statistically significant between T0 and T1 BA‐treated samples. The pathway of fatty acid salvage and aerobic respiration I (cytochrome c) increased, whereas TCA cycle I and TCA cycle V (2‐oxoglutarate: ferredoxin oxidoreductase) decreased as a consequence of BA administration (Supporting Information S1: Figure S8).

### 3.4 Statistically significant SCFAs from targeted metabolomics

Welch FDR corrected test merged with a fold change analysis allowed for ascertaining that all the five SCFA concentrations significantly changed between BA‐treated samples (T1) and T0 (Supporting Information S1: Table S2). Specifically, isovaleric acid increased in BA‐treated samples, whereas propanoic, acetic and butanoic acids decreased in their normalized ppm concentrations (Supporting Information S1: Figure S9).

## 4 DISCUSSION

For the first time, we here demonstrate the butyrate effectiveness on abdominal pain relief in an IBS pediatric patient cohort.

The therapeutic potential of butyrate in managing IBS has already been evaluated in adults. One of the earliest studies by Banasiewicz and colleagues investigated the effects of microencapsulated butyrate. In their double‐blind, randomized controlled trial the authors demonstrated a significant reduction in the frequency of abdominal pain and improvements in symptoms such as postprandial pain and urgency after 12 weeks of treatment.^13^

Lewandowski et al. expanded on this study by conducting a large multicenter cross sectional trial on 2990 IBS patients. The study reported a statistically significant reduction in a broad range of symptoms, including abdominal pain, flatulence, diarrhea, and constipation, after 12 weeks of treatment with a patented microencapsulated butyrate preparation. In addition to symptom relief, the treatment significantly improved patients' QoL, social functioning, and professional work. Notably, 93.9% of the participants expressed willingness to continue the therapy, demonstrating the acceptability and feasibility of butyrate as a treatment option for IBS.^14^

Building on these findings, Gasiorowska et al. proposed a novel approach combining butyrate with probiotics and prebiotics.^15^ Their randomized, double‐blind, placebo‐controlled trial aims to evaluate the efficacy of a multi‐component intervention containing butyrate, probiotics (Lactobacillus and Bifidobacterium strains), and short‐chain fructooligosaccharides (scFOS) involving 120 IBS patients. The study demonstrated a significantly higher proportion of patients in the treatment group reporting adequate relief of symptoms at Week 4 (64.7% vs. 42.0%, *p* = 0.023) and a reduced rate of symptom worsening by Week 12 (5.9% vs. 16.0%, *p* = 0.015). While improvements in global symptom severity and QoL did not differ significantly from the placebo, the intervention effectively reduced the urgency to defecate.^15^

Although not directly comparable due to differences in study design, formulations used, and clinical scoring systems adopted, collectively, these studies highlight the potential of BA in alleviating IBS symptoms and improving QoL. However, it is crucial to note that all these trials were conducted exclusively in adult populations. Evidence on the efficacy and safety of butyrate in children with IBS remains limited, representing a significant literature gap.

In our study, the treatment was well tolerated, and no adverse events or side effects were reported in either the butyrate or placebo groups, confirming its safety in the pediatric population. This finding aligns with adult studies,^13^, ^14^, ^15^ which consistently reported good tolerability and absence of significant side effects following butyrate supplementation, either alone or in combination with biotics.

Findings in the present study demonstrate that butyrate supplementation leads to significant clinical improvement in over 70% of pediatric IBS patients, accompanied by marked changes in gut microbiota composition and metabolic profiles, suggesting potential mechanisms underlying its therapeutic effects.

Specifically, butyrate administration significantly enhanced the abundance of bacterial taxa implicated in the production of SCFAs and maintenance of gut homeostasis. Among these, the *Lachnospiraceae NC2004 group* exhibited a significant increase, consistent with its established role in fermenting complex carbohydrates to produce SCFAs^27^ maintaining epithelial barrier integrity, and modulating inflammatory responses. Similarly, *Clostridia UCG‐014* and the *R. gauvreauii* group, known for their capacity to degrade dietary fibers and generate SCFAs, were enriched following butyrate treatment. These microbiota changes align with improved gut barrier function and immune homeostasis, likely contributing to the clinical benefits observed.

Conversely, BA supplementation concurrently reduced the abundance of specific bacterial taxa associated with gut dysbiosis and inflammation. A notable decrease was observed in *Sellimonas*, a genus previously linked to altered metabolic pathways and prevalent in IBS patients,^28^ suggesting a beneficial shift toward microbial equilibrium. A decreased relative abundance for *Candidatus Saccharibacteria* (TM7 bacterial phylum) was detected in butyrate treated samples at T1. Initially found in the human oral niche^29^ a recently carried out taxonomic analyses revealed a higher diversity of TM7 phylotypes in Crohn disease patients if compared with ulcerative colitis and non‐IBD controls.^30^ Additionally, a significant reduction in *R. gnavus*, recognized for its role in pro‐inflammatory polysaccharide production and association with inflammatory conditions such as Crohn's disease,^31^ underscores the potential anti‐inflammatory impact of butyrate. Furthermore, the reduction of Saccharimonadaceae, characterized by an epibiotic and potentially disruptive ecological role,^32^ further supports the beneficial microbial shifts induced by supplementation.

Metabolomic analyses demonstrated marked changes in VOC and SCFA profiles. Decreases in pentanoic acid, benzyl alcohol, and acetonitrile indicate modifications in microbial fermentation pathways, particularly those involved in nitrogenous and lipid metabolism. Significant reductions in 1‐undecanol, 1‐undecene, and 9‐octadecene further suggest a microbial shift toward a more stable, anti‐inflammatory gut environment. The observed increase in octanoic acid, a medium‐chain fatty acid with known antimicrobial properties,^33^ further implies an enhanced microbial activity conducive to gut homeostasis.

Targeted SCFA analysis reinforced these metabolic shifts, showing elevated levels of isovaleric acids, indicative of increased branched‐chain amino acid fermentation and a potential shift towards protein metabolism.^34^ Conversely, reductions in propionate, acetate, and endogenous butyrate levels suggest modulation of carbohydrate fermentation pathways, likely influenced by the exogenous supply of butyrate through supplementation. This observation highlights the complex and dynamic interplay between exogenous supplementation and endogenous microbial metabolism.

Further longitudinal studies are essential to fully elucidate these mechanisms, evaluate long‐term outcomes, and refine supplementation strategies for optimal clinical application in pediatric IBS management. Collectively, these findings underscore the therapeutic potential of BA supplementation for pediatric IBS, highlighting complex interactions between microbiota composition, metabolic function, and clinical symptomatology.

The active formulation used in our study contained BA along with zinc and vitamin D, which may have contributed to the observed effects. Zinc plays an important role in maintaining intestinal barrier function and immune modulation,^35^ and reduced zinc levels have been specifically associated with IBS‐D.^36^ At date, no interventional studies have evaluated zinc supplementation as a treatment for IBS yet. As for vitamin D, a recent meta‐analysis showed that its supplementation can significantly improve symptom severity and QoL in patients with IBS, with a low risk of adverse effects.^37^ The inclusion of these micronutrients in the active formulation may have enhanced the clinical and microbiota‐related outcomes observed, potentially through synergistic mechanisms with butyrate.

The main limitations of the present study are related to (1) the small number of children recruited, (2) the availability of fecal samples for metabolomic analysis, (3) the short duration of the follow‐up period, which does not allow us to determine whether the observed treatment effects are sustained over time after discontinuation of butyrate supplementation, (4) the challenge of extending the treatment to younger children due to the requirement for tablet formulation and the difficulty in swallowing them.

Butyrate has a very pungent odor, which makes the oral intake unpleasant; moreover, once ingested, it is rapidly absorbed in the upper part of the GI tract, an event that considerably reduces its positive effects in the colon.

To overcome this limitation, tablet formulation is needed, formulated in a double‐layer pharmaceutical form with functional release that promote a protracted release of butyrate throughout intestinal transit.

Long‐term follow‐up studies, including re‐evaluation of the same cohort, are needed to confirm the durability of clinical improvement and to explore sustained microbiota and metabolic changes after butyrate supplementation.

## 5 CONCLUSION

In conclusion, this randomized clinical trial represents the first investigation into the efficacy of BA supplementation as an adjunctive therapeutic strategy for pediatric IBS. The results provide compelling evidence that BA supplementation significantly reduces abdominal pain and overall symptom severity. Additionally, multiomics analyses, encompassing metataxonomics, and targeted/untargeted metabolomics, reveal a notable enhancement in gut microbiota composition characterized by an increase in SCFA‐producing bacteria, a concurrent reduction in pro‐inflammatory taxa, and beneficial metabolic shifts promoting gut homeostasis. These microbiome alterations likely underpin the clinical improvements observed in IBS symptoms.

Although these findings represent an important advancement in pediatric gastroenterology, further large‐scale clinical studies are necessary to confirm the therapeutic benefits observed, elucidate the underlying mechanisms comprehensively, and establish standardized dosing protocols and patient‐friendly formulations specifically suited to pediatric patients.

## CONFLICT OF INTEREST STATEMENT

The authors declare no conflicts of interest.

## Supporting information

Supporting information.
