# Supplementary material for: Calcium butyrate efficacy in pediatric irritable bowel syndrome: Randomized placebo‐controlled multiomics‐based clinical trial
Source: J Pediatr Gastroenterol Nutr. 2025 Jul 9;81(3):551–61. doi: 10.1002/jpn3.70154 (PMC12408980; doi:10.1002/jpn3.70154)

Supplementary Table S1: Symptomatic scores at baseline (T0), end of treatment (T1) and end of follow up (T2)

	VAS TO		GSRS TO		VAS T1		GSRS T1		VAS T2		GSRS T2	
	Butyrate	Placebo	Butyrate	Placebo	Butyrate	Placebo	Butyrate	Placebo	Butyrate	Placebo	Butyrate	Placebo
Patients (n)	25	25	25	25	25	25	25	25	25	25	25	25
Mean	6.07	4.64	13.92	12.48	2.07	4.36	6.88	11.00	1.23	3.17	5.44	8.52
95% CI Upper	6.60	5.18	15.05	14.27	2.67	4.96	8.55	13.25	1.71	3.96	7.16	10.39
95% CI Lower	5.54	4.09	12.78	10.68	1.46	3.76	5.21	8.74	0.76	2.38	3.71	6.64
Std. Deviation	1.28	1.31	2.75	4.35	1.47	1.45	4.04	5.47	1.14	1.91	4.17	4.54

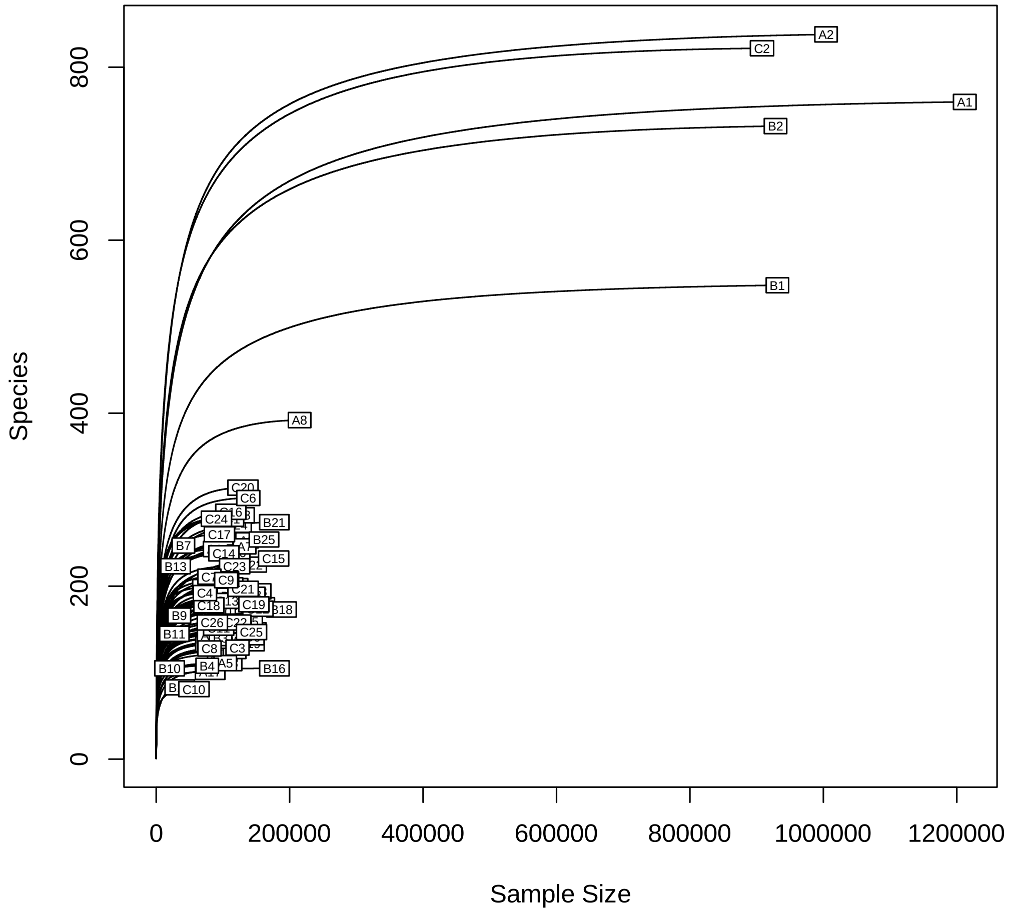

Supplementary Figure S1. Sample rarefaction curve. Sample size versus number of species identified in the IBS sample cohort.

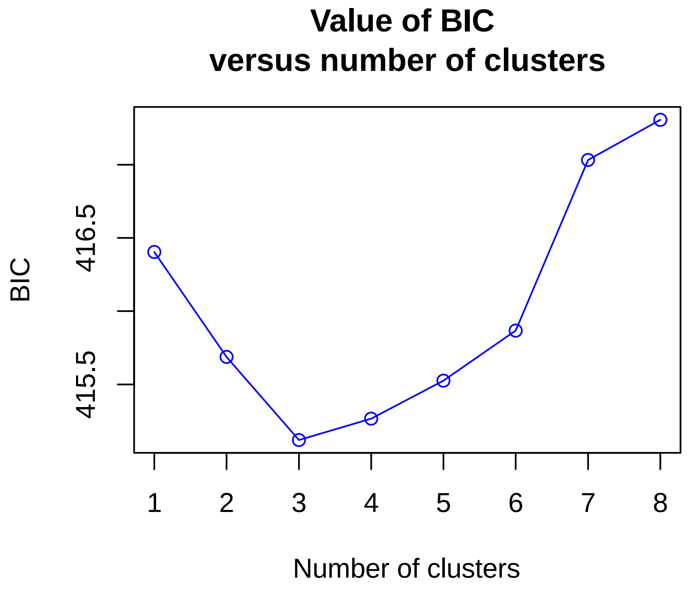

Supplementary Figure S2: optimal number of cluster (the elbow in the curve) inspected by Bayesian inference curve BIC curve where k-means analysis is run sequentially at increasing k values.

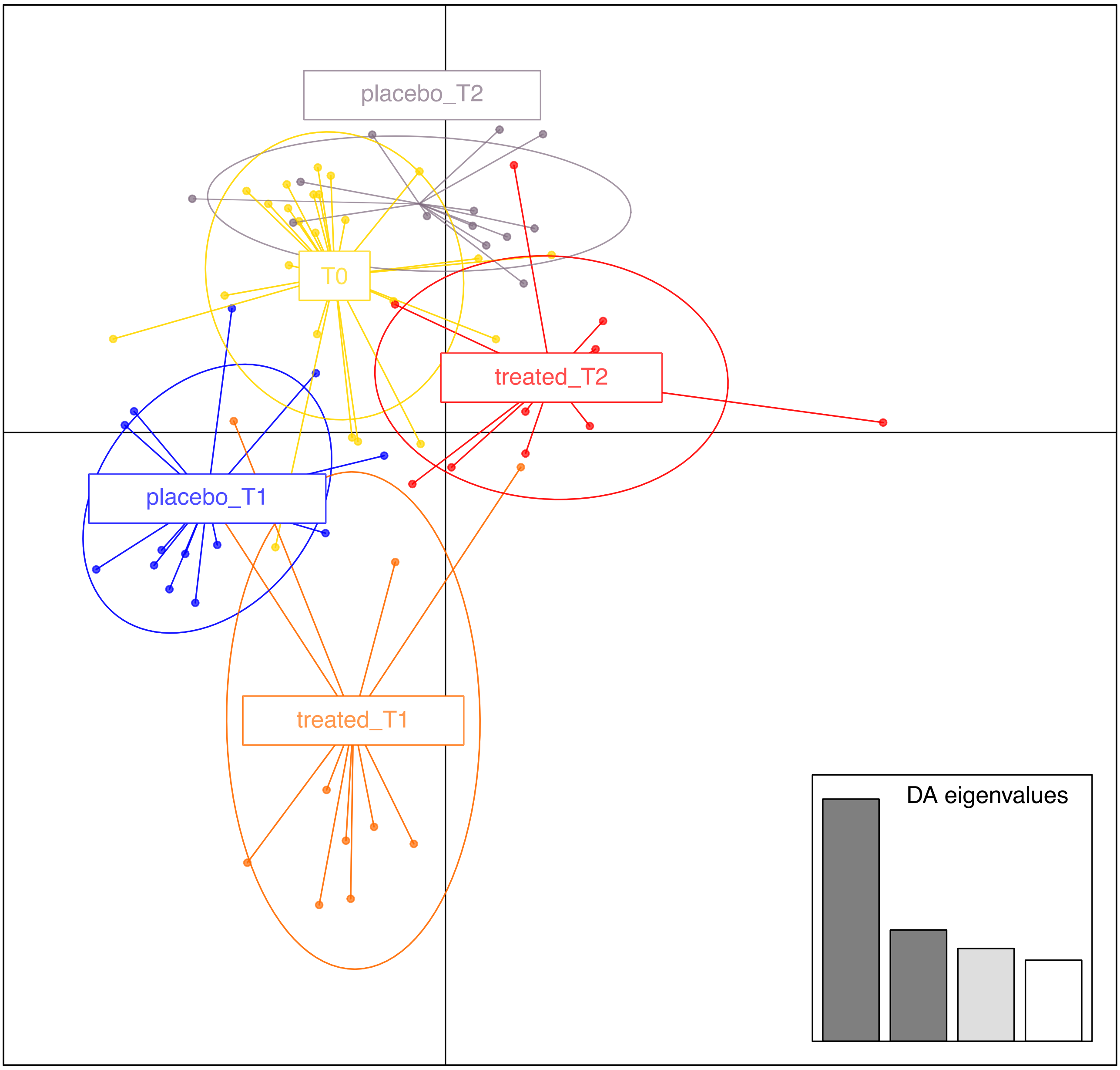

Supplementary Figure S3. A posterior DAPC plot. DAPC clusters obtained by retaining 60 principal components and 3 eigenvalues (discriminant function) corresponding to the variance ratio between groups over the variance within groups. The complete matrix of genus abundance was used as input file for computing the impact of eigen values. Different colours have been assigned at each group in the plot.

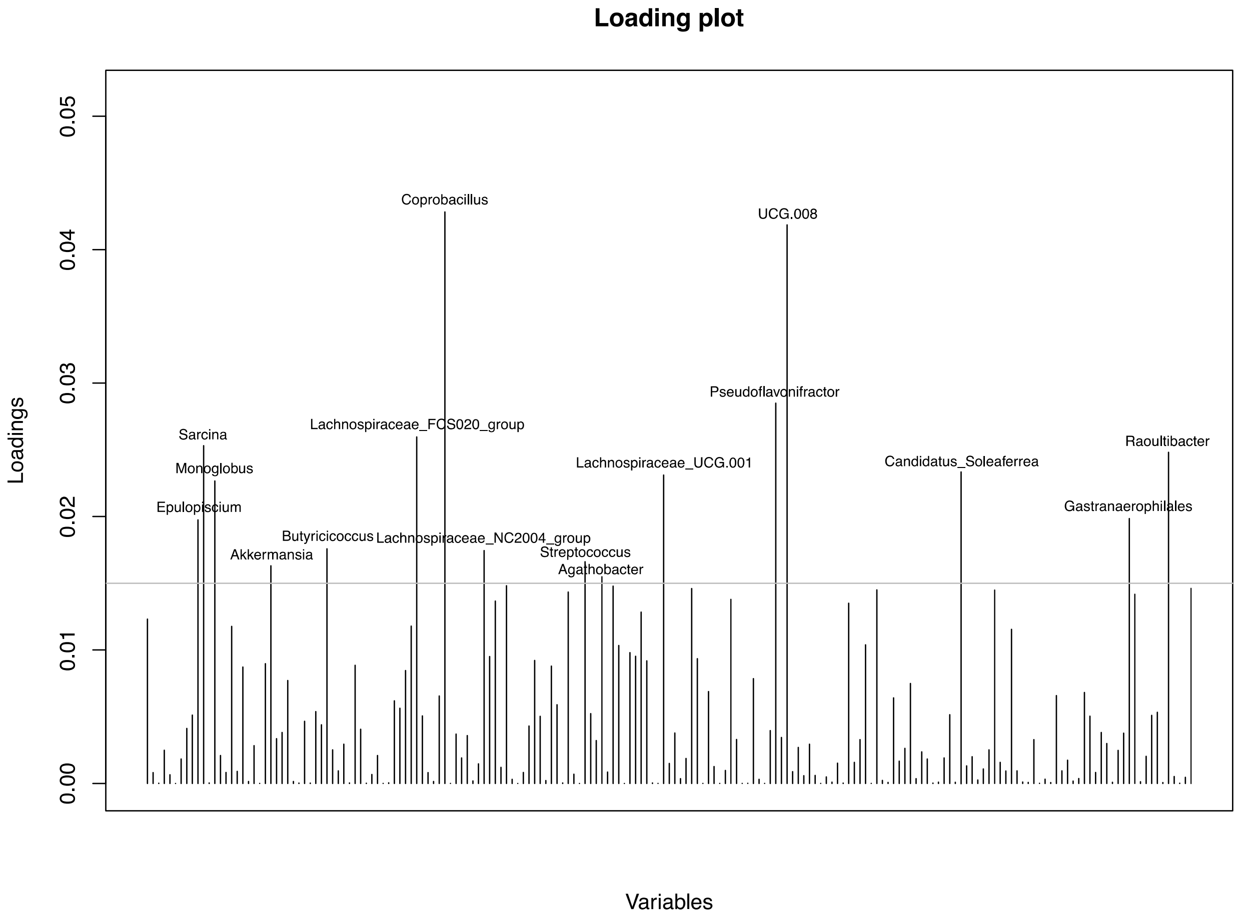

Supplementary Figure S4. DAPC assign plot. *A priori* and *posterior* group assignment probabilities collapsed in a matrix reporting for each sample the matching between the unsupervised and forced group assignments. Heat colours, from pale yellow to red represent a scale membership probability (red=1, white=0); blue crosses represent the prior cluster provided to DAPC. In most of samples DAPC classification is consistent with the a priori cluster assignment.

Supplementary table S2. Volatile organic compound (VOC) profiles

Compounds	Classes
Ethanol	Alcohols
1-Butanol, 3-methyl-	Alcohols
1-Octanol	Alcohols
1-Hexanol	Alcohols
2-Decanol	Alcohols
Menthol	Alcohols
2-Undecanol	Alcohols
Benzyl Alcohol	Alcohols
1-Dodecanol	Alcohols
Phenylethyl Alcohol	Alcohols
1-Undecanol	Alcohols
1-Butanol	Alcohols
Ethanol, 2-butoxy-	Alcohols
Acetaldehyde	Aldehydes
Propanal, 2-methyl-	Aldehydes
Butanal, 3-methyl-	Aldehydes
Nonanal	Aldehydes
Methional	Aldehydes
Pentadecanal-	Aldehydes
2,4-Decadienal, (E,E)-	Aldehydes
Benzaldehyde, 2,5-dimethyl-	Aldehydes
Butanal	Aldehydes
Benzaldehyde, 4-hydroxy-	Aldehydes

Compounds	Classes
Butanoic acid, ethyl ester	Carboxylic Acids
Acetic acid	Carboxylic Acids
Propanoic acid	Carboxylic Acids
Propanoic acid, 2-methyl-	Carboxylic Acids
Butanoic acid	Carboxylic Acids
1-Hexadecanol	Carboxylic Acids
Butanoic acid, 3-methyl-	Carboxylic Acids
Butanoic acid, 2-methyl-	Carboxylic Acids
Pentanoic acid	Carboxylic Acids
Heptanoic acid	Carboxylic Acids
Cyclohexene, 1-methyl-4-(1-methylethenyl)-, (S)-	Carboxylic Acids
Octanoic Acid	Carboxylic Acids
1H-Pyrrole-2,5-dione, 3-ethyl-4-methyl-	Carboxylic Acids
1H-Pyrrole-2,5-dione, 3-ethenyl-4-methyl-	Carboxylic Acids
Dodecanoic acid	Carboxylic Acids
Ethyl Acetate	Carboxylic Acids
Propanoic acid, ethyl ester	Carboxylic Acids
Butanoic acid, methyl ester	Carboxylic Acids
Butanoic acid, propyl ester	Carboxylic Acids
Butanoic acid, butyl ester	Carboxylic Acids
Hexanoic acid, ethyl ester	Carboxylic Acids
Hexanoic acid, propyl ester	Carboxylic Acids
Hexanoic acid, butyl ester	Carboxylic Acids
Butanoic acid, hexyl ester	Carboxylic Acids
Butanoic acid, 3-methylbutyl ester	Carboxylic Acids
Benzeneacetic acid, methyl ester	Carboxylic Acids
Benzeneacetic acid, ethyl ester	Carboxylic Acids
Hexanoic acid	Carboxylic Acids
Benzenepropanoic acid, ethyl ester	Carboxylic Acids
Cyclohexanecarboxylic acid	Carboxylic Acids
Eugenol	Carboxylic Acids
Benzenepropanoic acid	Carboxylic Acids
Formic acid	Carboxylic Acids
Pentanoic acid, ethyl ester	Carboxylic Acids
Pentanoic acid, propyl ester	Carboxylic Acids
Butanoic acid, pentyl ester	Carboxylic Acids
Pentanoic acid, 3-methylbutyl ester	Carboxylic Acids
Pentanoic acid, pentyl ester	Carboxylic Acids
Propanoic acid, 2-methyl-, propyl ester	Carboxylic Acids
Propanoic acid, 2-methylpropyl ester	Carboxylic Acids
Butanoic acid, 3-methyl-, propyl ester	Carboxylic Acids
Propanoic acid, pentyl ester	Carboxylic Acids
Butanoic acid, 3-methyl-, butyl ester	Carboxylic Acids
Hexanoic acid, methyl ester	Carboxylic Acids
Propanoic acid, propyl ester	Carboxylic Acids

Compounds	Classes
Acetic acid ethenyl ester	Carboxylic Ester
Benzenepropanoic acid, methyl ester	Carboxylic Ester
2-Methoxy-4-vinylphenol	Fatty acid
n-Hexadecanoic acid	Fatty Acid
Undecane, 3,8-dimethyl-	Hydrocarbons
9-Octadecene, (E)-	Hydrocarbons
Tetradecane	Hydrocarbons
Benzene, 1,3-bis(1,1-dimethylethyl)-	Hydrocarbons
1-Tetradecene	Hydrocarbons
1-Tridecene	Hydrocarbons
Acetone	Hydrocarbons
Phenol	Hydrocarbons
2,4-Di-tert-butylphenol	Hydrocarbons
Cyclopentane	Hydrocarbons
Cyclotetradecane	Hydrocarbons
Tridecane	Hydrocarbons
beta.-Bisabolene	Hydrocarbons
Phenol, 2-methyl-	Hydrocarbons
Phenol, 2-methyl-5-(1-methylethyl)-	Hydrocarbons
Hexadecane	Hydrocarbons
1-Hexadecene	Hydrocarbons
Octadecane	Hydrocarbons
Phenol, 2,3,5-trimethyl-	Hydrocarbons

Compounds	Classes
Indole	Indoles
1H-Indole, 3-methyl-	Indoles
1H-Indole, 5-methyl-	Indoles
1H-Indole, 2-methyl-	Indoles
2-Butanone	Ketones
Methyl Isobutyl Ketone	Ketones
5-Hepten-2-one, 6-methyl-	Ketones
2-Nonanone	Ketones
2-Decanone	Ketones
2-Undecanone	Ketones
2-Dodecanone	Ketones
Ethanone, 1-(3-aminophenyl)-	Ketones
2-Hexanone	Ketones
gamma-Dodecalactone	Lactones
Hexane, 2,3,4-trimethyl-	Others
Trimethylamine	Others
Methane, isocyanato-	Others
2-n-Propyl-1-heptanol	Others
Benzaldehyde, 4-propyl-	Others
Benzeneacetaldehyde, .alpha.-ethylidene-	Others
2-Pyrrolidinone, 1-methyl-	Others
n-Decanoic acid	Others
Acetonitrile	Others
Ethanone, 1-(2-furanyl)-	Others
1H-Indole, 2,3-dihydro-4-methyl-	Others
Dimethyl ether	Others
Dimethyl trisulfide	Others
Pyrazine, tetramethyl-	Others
Dimethyl sulfide	Others
Hydrazine, 1,2-dimethyl-	Others
Anethole	Others
Methyl isovalerate	Others
1-Butene, 4-isothiocyanato-	Others
1-Undecene, 7-methyl-	Others
Caryophyllene	Terpenes
2,6-Octadienal, 3,7-dimethyl-, (E)-	Terpenes
5,9-Undecadien-2-one, 6,10-dimethyl-, (E)-	Terpenes
Bicyclo[3.1.0]hexane, 4-methylene-1-(1-methylethyl)-	Terpenes
Humulene	Terpenes
alph.-Muurolene	Terpenes
Camphene	Terpenes

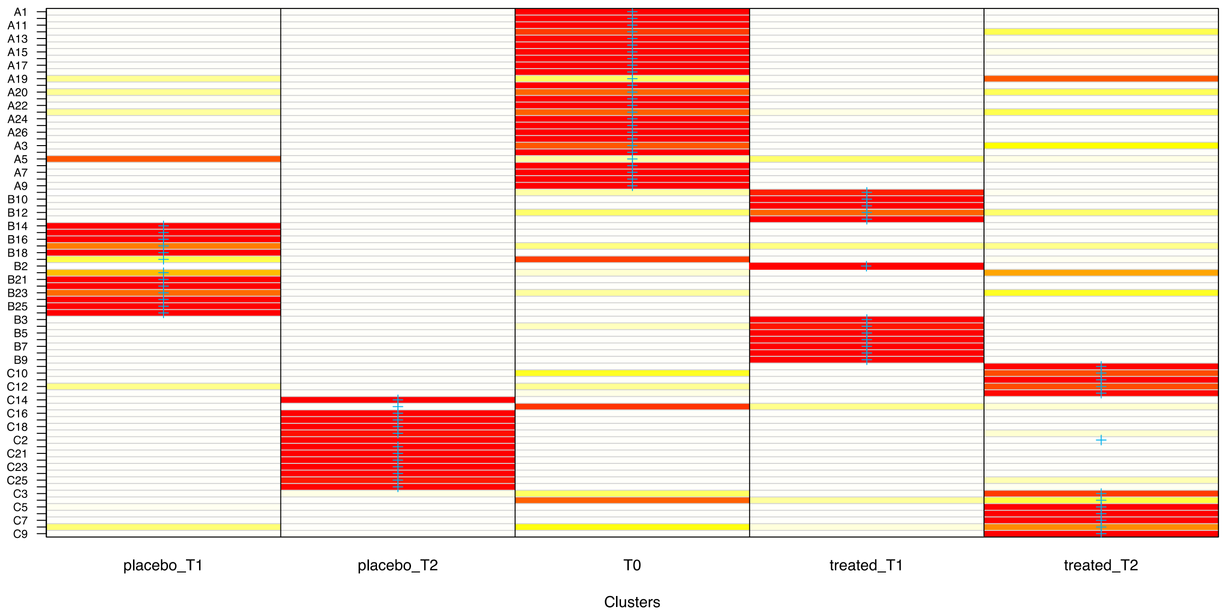

Supplementary Figure S5. DAPC loading plot. Variable loading greater than 0.015 was used as an arbitrary threshold to select those genera (sixteen in total) that much more contributed to the DAPC cluster separation.

Supplementary Table S3. Targeted metabolomics SCFA average values and standard deviation. Basic statistics on SCFA (acetate, propanoate, isobutyrate, butanoate and isovalerate) detected average ppm values and relative Avg. = average; Std. dev. = standard deviation.

	**Placebo Run-in**		**Placebo Treatment**		**Placebo Washout**		**BA Run-in**		**BA Treatment**		**BA Washout**	
**SCFA**	**Avg.**	**Std. dev.**	**Avg.**	**Std. dev.**	**Avg.**	**Std. dev.**	**Avg.**	**Std. dev.**	**Avg.**	**Std. dev.**	**Avg.**	**Std. dev.**
**Acetic acid**	2.0006	0.7899	2.3808	0.9785	2.1694	0.4820	2.0642	1.2064	3.2171	1.7937	1.3155	0.5856
**Propanoic acid**	1.1890	0.4694	1.4149	0.5815	1.28938	0.2865	1.2268	0.7170	1.9120	1.0660	1.3155	0.5856
**Isobutyric acid**	1.6393	0.6472	1.9508	0.8018	1.7776	0.3950	1.6914	0.9885	2.6361 6	1.4698	1.3155	0.5856
**Butanoic acid**	2.7239	1.0755	3.2416	1.3323	2.9539	0.6563	2.8105	1.6426	4.3803	2.4422	1.3155	0.5856
**Isovaleric acid**	3.0816	1.2167	3.6672	1.5072	3.3417	0.7425	3.1795	1.8583	4.9554	2.7629	1.3155	0.5856

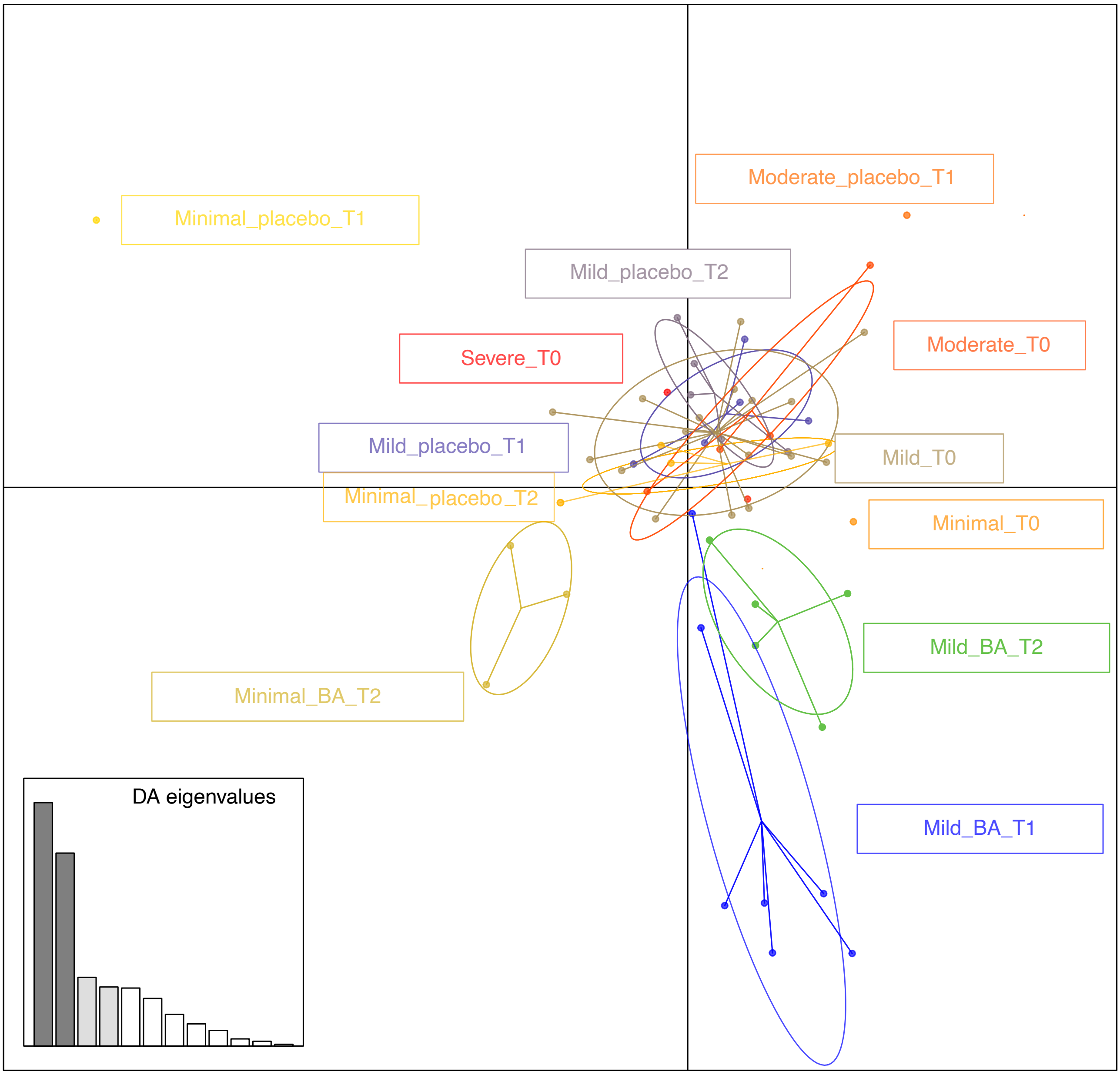

Supplementary Figure S6. DAPC plot based on GSRS scale classes and timing. The evaluation of GSRS symptoms based on patient administered questionnaires has been used to a posterior cluster sample. Patients sampling occurred before treatment, after 8 weeks (T1) and after 4 weeks (T2 washout). 60 principal components and 4 eigen values have been used to scatter the DAPC plot.

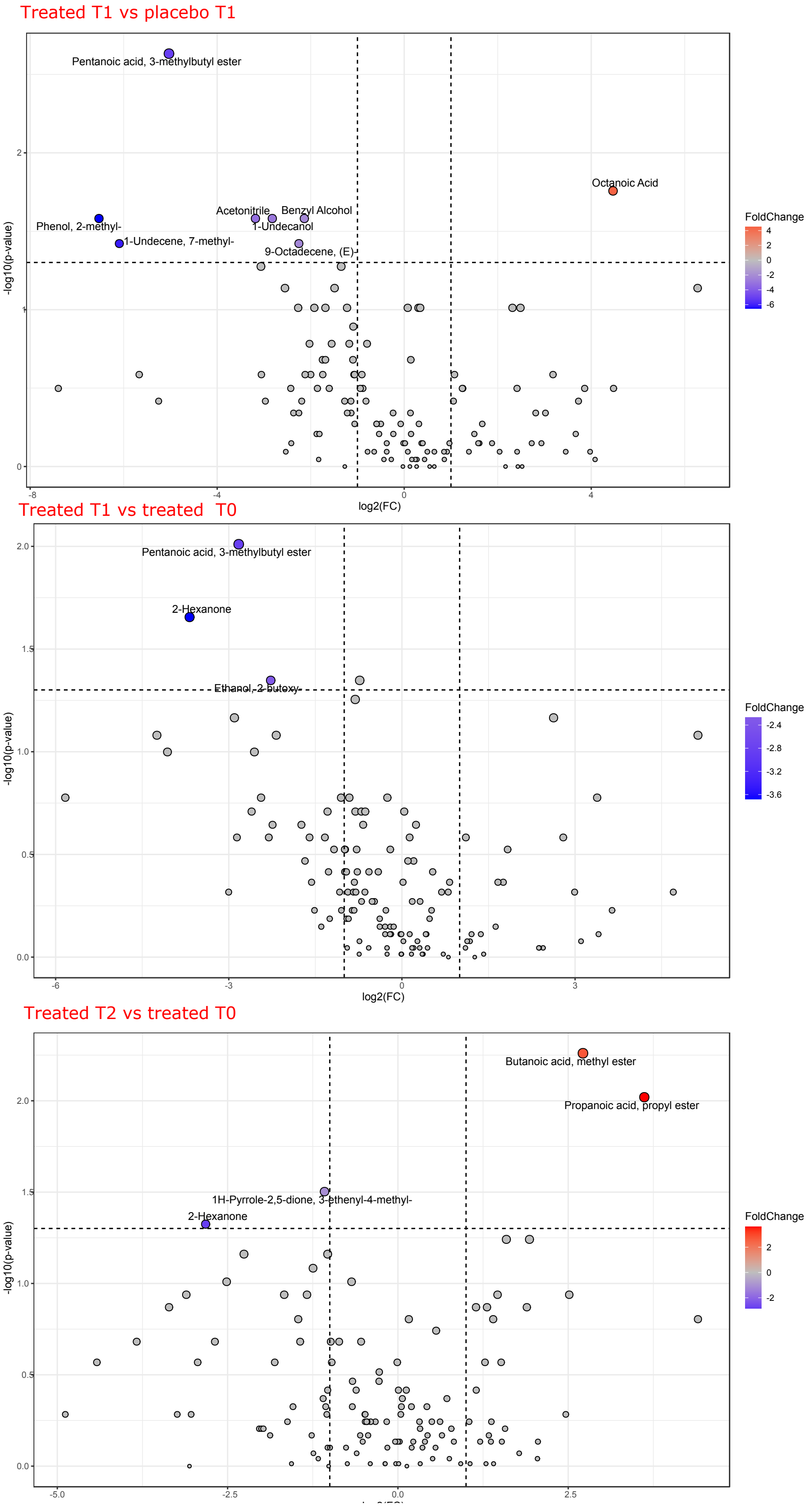

Supplementary Figure S7. Volcano plot of statistically significant metabolites. A Welch's test and a Fold Change (FC) analysis were used to infer statistically significant VOCs from metabolomics. The three plots report: i) the comparison between placebo T1 and BA treated (T1) samples, ii) untreated samples in the BA arm versus BA treated (T1) samples, iii) untreated samples in the BA arm versus BA samples at washout (T2).

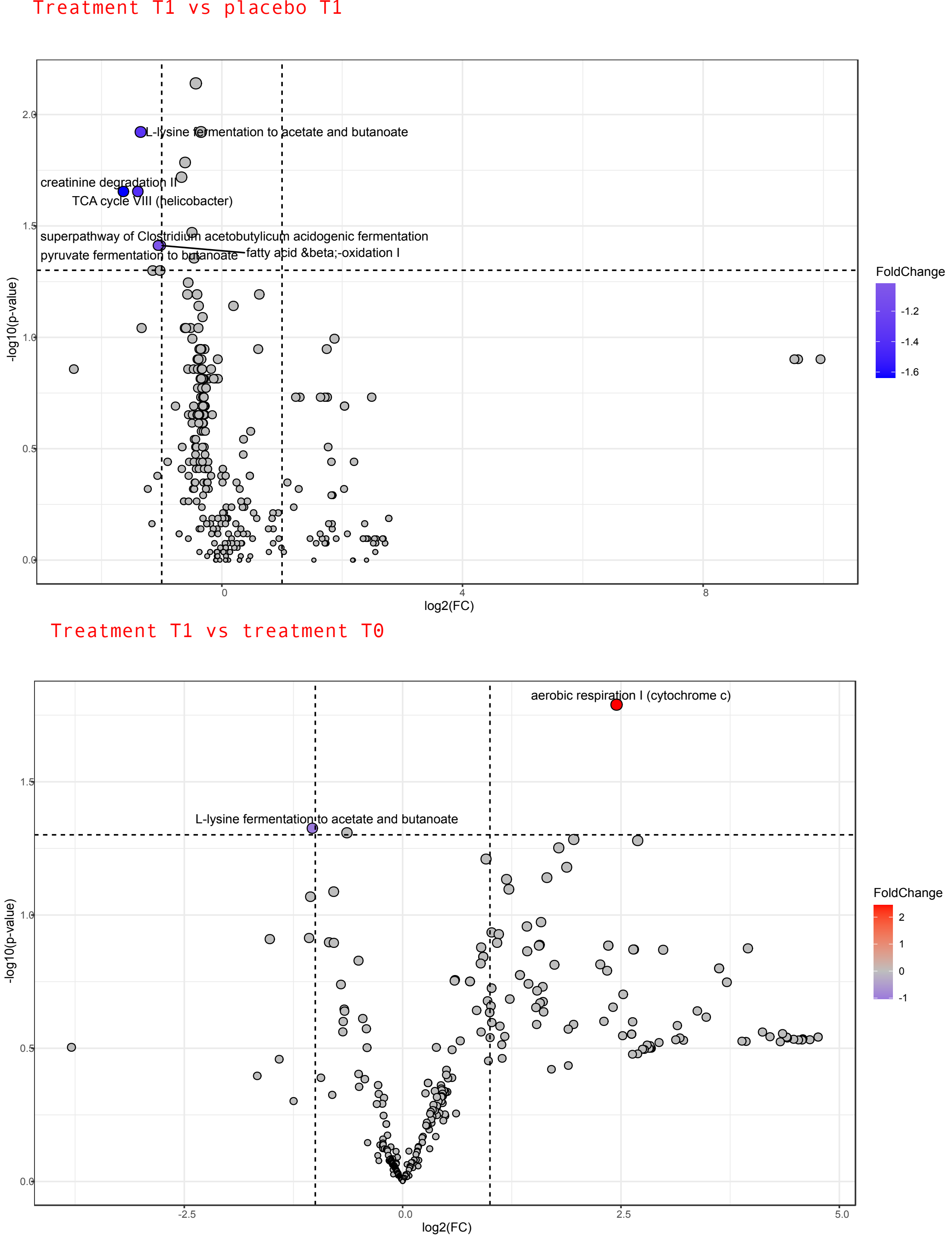

Supplementary Figure S8. Statistically significant metabolic biochemical pathways from 16S matrix prediction. The two volcano plots report the statistically significant biochemical pathways derived from a Welch test combined with a fold change (FC) analysis corrected by FDR. Panel A) placebo versus treated samples at T1. Increased or decreased FC values are relative to the first term in the comparison, i.e. placebo T1. Panel B) BA treated at T1 versus untreated (T0) in the CB arm. Increased or decreased FC in violet or blue colour respectively are relative to the first term in the comparison, i.e. untreated in the CB arm.

Supplementary Figure S9. SCFA volcano plot and single SCFA distribution violin plots. Welch's test FDR adjusted analysis joined with a fold change (FC) analysis is reported as volcano plot precisely indicating increased (red) or decreased (violet) SCFA in BA treated (T1) versus untreated samples.

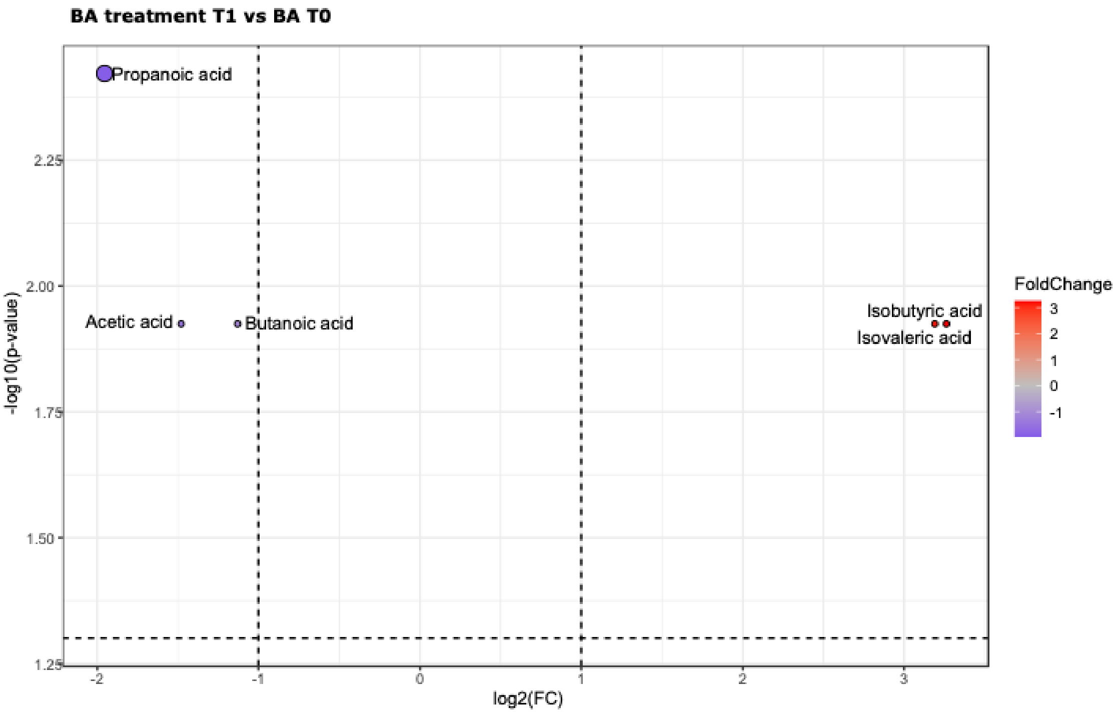

SCFA	FC	log2 FC	p.ajusted	-LOG10(p)
Propanoic acid	0.2580	-1.9541	0.0037851	2.4219
Isobutyric acid	9.5937	3.2621	0.011891	1.9248
Isovaleric acid	9.1273	3.1902	0.011891	1.9248
Acetic acid	0.3585	-1.48	0.011891	1.9248
Butanoic acid	0.4570	-1.1297	0.011891	1.9248

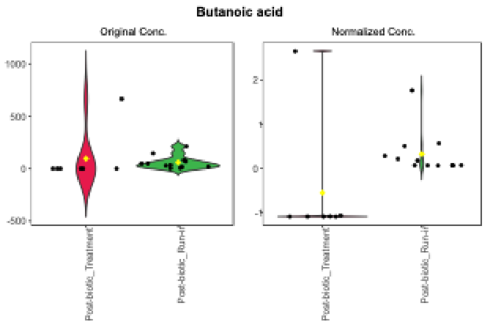

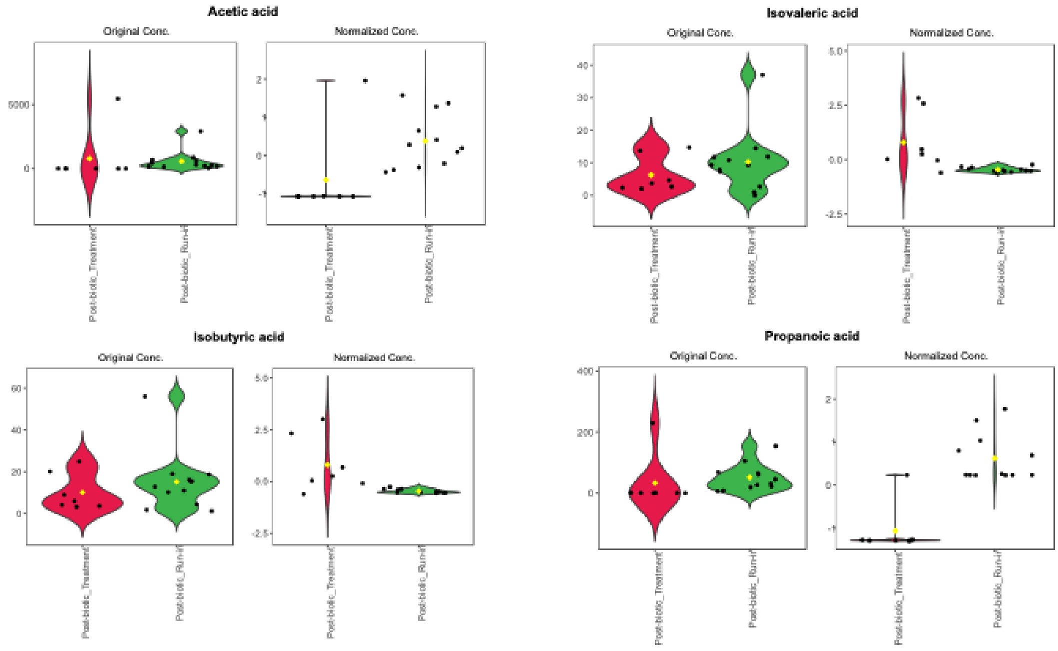

Supplement: Supplementary file 1 — Supporting information. [file JPN3-81-551-s001.pdf]
